# Brain uptake pharmacokinetics of albiglutide, dulaglutide, tirzepatide, and DA5-CH in the search for new treatments of Alzheimer’s and Parkinson’s diseases

**DOI:** 10.1080/21688370.2023.2292461

**Published:** 2023-12-14

**Authors:** Elizabeth M. Rhea, Alice Babin, Peter Thomas, Mohamed Omer, Riley Weaver, Kim Hansen, William A. Banks, Konrad Talbot

**Affiliations:** a Veterans Affairs Puget Sound Health Care System, Geriatrics Research Education and Clinical Center, Seattle, WA, USA; b Division of Gerontology and Geriatric Medicine, Department of Medicine, University of Washington School of Medicine, Seattle, WA, USA; c Departments of Neurosurgery, Pathology and Human Anatomy, and Basic Sciences, Loma Linda University School of Medicine, Loma Linda, CA, USA

**Keywords:** Alzheimer’s disease, glucagon-like peptide -1 receptor, incretin receptor agonists, Parkinson’s disease, pharmacokinetics

## Abstract

**Background:**

A number of peptide incretin receptor agonists (IRAs) show promise as therapeutics for Alzheimer’s disease (AD) and Parkinson’s disease (PD). Transport across the blood–brain barrier (BBB) is one way for IRAs to act directly within the brain. To determine which IRAs are high priority candidates for treating these disorders, we have studied their brain uptake pharmacokinetics.

**Methods:**

We quantitatively measure the ability of four IRAs to cross the BBB. We injected adult male CD-1 mice intravenously with ^125^I- or ^14^C-labeled albiglutide (biological active fragment, BAF), dulaglutide (BAF), DA5-CH, or tirzepatide and used multiple-time regression analyses to measure brain kinetics up to 1 hour. For those IRAs failing to enter the brain 1 h after intravenous injection, we also investigated their ability to enter over a longer time frame (i.e., 6 h).

**Results:**

Albiglutide (BAF) and dulaglutide (BAF) had the fastest brain uptake rates within 1 hour. DA5-CH appears to enter the brain rapidly, reaching equilibrium quickly. Tirzepatide does not appear to cross the BBB within 1 h after iv injection but like albumin, did so slowly over 6 h, presumably via the extracellular pathways.

**Conclusions:**

We find that IRAs can cross the BBB by two separate processes; one that is fast and correlates to lipid solubility and one that is slow and is related to acylation. Three of the four IRAs investigated here have fast rates of transport and should be taken into consideration for testing as AD and PD therapeutics as they would have the ability to act quickly and directly on the brain as a whole.

## 1. Introduction

The Aβ antibodies lecanemab (Leqembi)^1^ and donanemab^2^ are the first Alzheimer’s disease (AD) therapeutics demonstrated in phase III trials to significantly reduce cognitive decline to a small degree that nevertheless appears clinically significant. Yet these therapeutics, like the Aβ antibody aducanumab, pose serious safety concerns since they risk amyloid-related imaging abnormalities (ARIAs), reflecting parenchymal vascular leakage^3^ and associated brain volume loss.^4^ Such risks may have contributed to several deaths reported in phase III trials of aducanumab,^5^ lecanemab,^6–8^ and donanemab^9^ antibodies.

Since phase III AD trials of drugs other than Aβ antibodies have failed,^10–12^ as have phase III trials on disease modifying treatments for PD,^13–16^ the wide-ranging search for new AD and PD treatments continues. Among the targets of the new treatments is brain insulin resistance,^17–22^ which is a common and prominent feature in AD^23^ and has now been shown in PD,^19,20^ specifically in cognitively impaired PD cases.^20,24^

Brain insulin resistance impairs neuronal survival and function in many ways by retarding such processes as anti-apoptosis, autophagy, adult dentate neurogenesis, and synaptic plasticity.^25–29^ Studies testing brain insulin resistance in animal models of PD are limited at present, but such resistance has been established in animal models of AD.^30,31^ Brain insulin resistance in these models can be induced by many AD risk factors^30,32–35^ and can in turn promote many AD cellular pathologies and cognitive deficits.^25,30,34–36^

Antidiabetic drugs known as incretin receptor agonists (IRAs) can substantially reduce brain insulin resistance and its associated molecular pathologies and cognitive deficits in animal models of AD and PD.^29,37,38^ There are three current classes of IRAs:^1^ peptide-based single agonists activating receptors for one of the two major incretin hormones, namely glucagon-like peptide-1 (GLP-1),^2^ peptide-based dual-agonists activating receptors for both major incretins (GLP-1 and glucose-dependent insulinotropic polypeptide [GIP]), and.^3^ non-peptide, small molecule and positive allosteric modulators of GLP-1 R.^39^ Thus far, only the first two of these classes, which we refer to as peptide IRAs, are reported to have beneficial effects on brain insulin resistance and animal models of AD and PD.^20,24,37,38,40–47^ The clinical importance of using peptide IRAs to treat disorders associated with cognitive decline is highlighted by recent reports that dementia risk is significantly reduced in those with type 2 diabetes (T2D) treated with peptide IRAs (albiglutide, dulaglutide, exenatide, liraglutide, or semaglutide), but less so with other antidiabetics.^48,49^

We previously investigated the BBB transport capabilities of nine IRAs found to be therapeutic in animal models of AD and PD and found that two of them (exenatide and an experimental dual IRA, DA4-JC) had the fastest transport rates.^50,^^a^aThe Finan and Ma et al. (2013) peptides studied in our earlier report^50^ were identified as Peptides 17, 18, and 20 based on numbering in Table 1 of the former paper. These are respectively unmodified, acylated, and PEGylated forms of dual IRAs. Our earlier numbering of the last two peptides is, however, ambiguous since Table 1 in Finan and Ma et al. (2013) lists two peptide 18s. Using the unambiguous numbering in supplementary figure 1 of Finan and Ma et al. (2013), the acylated and PEGylated dual IRAs we studied are Peptides 19 and 21, respectively. Peptide 17, the unmodified dual IRA of Finan and Ma et al. (2013) is the backbone of all other current dual IRAs except tirzepatide. Christian Hölscher’s group refers to these peptides and their variants using a nomenclature starting with DA for dual agonist and ending with CH (Chinese) or JC (Japanese) for their manufacturing source. The relationship between the dual IRAs identified by the two research groups (and clinical studies) is as follows. Peptide 18 (which differs from Peptide 17 only by a lysine substitution for the terminal cysteine) is DA3-CH.^38,62^ Peptide 18 with a 5-lysine C-terminal extension is DA4-JC.^38,62^ Peptide 18 with an RRQRRKKRGY C-terminal extension is DA5-CH (= DA-CH5).^38,62^ Peptide 19 (the acylated dual IRA) is DA1-JC (= DA-JC1),^38,62^ NNC0090 of Frias et al.,^88^ and MAR709 of Novikoff et al.^89^ Peptide 21 (the PEGylated dual IRA) is DA2.^62^ Since that publication, we identified four additional IRAs worthy of investigation for BBB transport: the FDA-approved single IRAs albiglutide (Tanzeum/Eperzan)^51–54^ and dulaglutide (Trulicity),^54–58^ the FDA-approved dual IRA tirzepatide (Mounjaro),^53,59–61^ and the experimental dual IRA DA5-CH originally named DA-CH5.^38,62^This last drug is especially potent in reducing neuropathology (e.g., neuron loss; decreased synaptic plasticity; elevated Aβ, hyperphosphorylated tau, and α-synuclein; neuroinflammation; and mitochondrial dysfunction), and cognitive deficits in animal models of AD and PD.^29,38^ In the cases of albiglutide and dulaglutide, biologically active fragments (BAFs) were tested. These IRAs might have faster BBB transport rates than previously investigated IRAs due to their structures.

Since substances that are extremely stable in blood, like many IRAs, potentially enter the brain slowly via the extracellular pathways outside the BBB,^63,64^ we also tested the possibility that IRAs are unable to accumulate significantly in the brain during our standard 1-hour protocol do so after a longer study period of 6 hours.

Throughout this report, we compare results on the four IRAs tested here with comparable results on the nine IRAs we tested earlier.^50^ In our Discussion, we consider all 13 IRAs and the literature on them to determine which of all these IRAs merit high priority for clinical trials in AD and PD based on the ability to cross the BBB.

## 2. Materials and methods

### 2.1. Peptide sources

All but one peptide (DA5-CH) was obtained from commercial sources: albiglutide (BAF) (TP1796) from TargetMol (Wellesley, MA), dulaglutide (BAF) (GC31520) from GLPBIO (Montclair, CA), liraglutide (6517) from Tocris Bioscience (Minneapolis, MN), semaglutide (B0084–007194) and tirzepatide (BAT-006246) from BOC Sciences (Shirley, NY). DA5-CH was custom synthesized for us by AnaSpec. Sequences and additional peptide information are listed in Table 1.

**Table 1.** Characteristics of the IRA peptides studied.

Peptide	Description^a^	Sequence and Amino Acid Number^b^	MW (Da)^c^	Charge^d^	Hydro phobic amino acids	Lipid Solubility Partition Coefficient	Log Partition Coefficient (Log P)
*Single IRAs:*							
**Albiglutide (BAF)**	Bioactive fragment of hGLP-1 (7-36) with the alanine at position 8 replaced with a glycine to enhance resistance to DPP-4	HGEGTFTSDVSSYLEGQAAKEFIAWLVKGR (30 aa)	3284	0.09	37%	0.0132 ± 0.0040	-1.916 ± 0.121
**Dulaglutide (BAF)**	Bioactive fragment of hGLP-1 (7-37) monomer with 3 aa substitutions	HGEGTFTSDVSSYLEEQAAKEFIAWLVKGGG (31 aa)	3315	-2.0	35%	0.0102 ± 0.0007	-1.991 ± 0.0287
*Dual IRAs:*							
**DA5-CH**	Balanced GIPR/GLP-1R agonist (Peptide 18 of Finan et al. [2013]) with an 11 amin acid N-terminal extension (KRRQRRKKRGY)	YXEGTFTSDYSIYLDKQAAXEFVNWLLAGGPSSGAPPPSKRRQRRKKRGY-NH2 (50 aa)	5,620	6	44%	0.0066 ± 0.0004	-2.183 ± 0.0281
**Tirzepatide**	GIPR biased GIPR/GLP-1R agonist acylated at lysine 26 to bind albumin	YXEGTFTSDYSIXLDKIAQKAFVQWLIAGGPSSGAPPPS (39 aa)	4,813	-1	32%	0.1318 ± 0.0126*	-0.884 ± 0.0405*

^a^hGLP-1R = human glucagon-like peptide 1 receptor; hGLP-1R/GIPR = human GLP-1R + human glucose dependent insulinotropic polypeptide (GIP) receptor; BAF = bioactive fragment; DPP-4 = dipeptidyl peptidase-4

^b^The sources for complete amino acid sequences of the IRAs listed are as follows: for albiglutide, the FDA Database (http://crdd.osdd.net/raghava/thpdb/display_thppid_sub.php?details=Th1161), for dulaglutide, the FDA Database: http://crdd.osdd.net/raghava/thpdb/display_thppid_sub.php?details=Th1176), for DA5-CH Hölscher (2018), and tizepatide (Coskun et al., 2018).

^c^Molecular weight (g/mol = Da)

^d^Net charge at pH 7 was determined using calculator at http://www.bachem.com/service-support/peptide-calculator/

* p < 0.05 compared to lipid solubility of all other IRAs tested

### 2.2. Animal use

All studies used male CD-1 mice (8–10 weeks old) purchased from Charles River Laboratories (Seattle, WA). Mice had *ad libitum* access to food and water and were kept on a 12 h light/12 h dark cycle. Mice were anesthetized with an intraperitoneal (ip) injection of 0.15 mL of 40% urethane before each study and kept on a heating pad to maintain normal body temperature. The Institutional Animal Care and Use Committee (IACUC) at the Veterans Affairs Puget Sound Health Care System (VAPSHCS) in Seattle, WA, approved all animal studies and took place in a facility approved by the Association for Assessment and Accreditation of Laboratory Animal Care International (AAALAC).

### 2.3. Radioactive labeling

Five of the IRAs (albiglutide (BAF), dulaglutide (BAF), liraglutide, semaglutide, and tirzepatide) were radioactively labeled with ^125^I as previously described.^50^ Ten micrograms of each IRA were labeled with 0.5–1 mCi Na ^125^I (Perkin Elmer, Waltham, MA) in a reaction containing 10 µg of chloramine-T (Sigma-Aldrich, St. Louis, MO) in 0.25 M chloride-free sodium phosphate buffer, pH 7.5 (PB). The reaction was terminated 1 min later by adding 100 µg of sodium metabisulfite (Sigma-Aldrich). Bovine serum albumin (BSA, Sigma-Aldrich) was labeled with ^99m^Technetium (^99m^Tc, RLS, Seattle, WA) by combining 1 mCi ^99m^Tc with 1 mg albumin, 120 µg stannous tartrate, and 20 µL 1 M HCl in 500 µL deionized water for 20 min. Radioactively labeled IRAs (^125^I-IRAs) and albumin (^99m^Tc-Alb) were purified on Sephadex G-10 columns (Sigma-Aldrich), collected in 1% BSA lactated Ringer’s solution (BSA/LR) and characterized by 15% trichloroacetic acid (TCA, Fisher Scientific) protein precipitation. Greater than 90% radioactivity in the precipitated fraction was consistently observed for the IRAs tested and albumin. Since we were unable to label DA5-CH with ^125^I as previously noted,^50^ we used the Fred Hutchinson Cancer Center (Seattle, WA) to radioactively label DA5-CH with ^14^C as previously described^65^ and based on the procedure of Jentoft and Dearborn.^66^ Briefly, 10 mg of IRA was solubilized in 1 ml of phosphate buffered saline (PBS). Approximately 3 mg IRA was recovered following labeling with a specific activity of 198 Ci mol^−1^. Purity was verified using HPLC.

### 2.4. Octanol/buffer partition coefficient

We measured the lipid solubility of the IRAs, using octanol and PB. Briefly, 1 × 10^5^ counts per minute (cpm) of each ^125^I/^14^C-IRA were added to triplicate tubes containing equal volumes PB and octanol (Sigma-Aldrich). This solution was vigorously mixed for 1 min and centrifuged at 5500 ×g for 10 min to separate the two phases. Aliquots of 100 μl were taken in duplicate from each phase, and total radioactivity was counted in a gamma counter (Wizard2, Perkin-Elmer) or beta counter (TriCarb 3110TR, Perkin-Elmer). The ratio of the cpm in the octanol phase to the cpm in the buffer phase equates to the mean partition coefficient (Pcoeff) and indicates lipophilicity. The log value for the coefficient can be either greater than unity, indicative of a lipophilic compound, or less than unity, indicative of a hydrophilic compound.

### 2.5. In vivo stability of ^125^I/^14^C-IRAs in brain and blood

We measured the stability of the radiolabeled IRAs in brain and blood. Following anesthetization, arterial blood and whole brain were taken at two time points (10 or 60 min) after iv injection of 3 × 10^5^ cpm-1×10^6^ cpm of ^125^I/^14^C-IRA in 0.2 ml of BSA/LR. The blood samples were allowed to clot, centrifuged at 3200 ×g for 10 min, and 50 μl of the separated serum was added to 250 μl of BSA-LR. Samples were combined with equal volume 30% TCA (300 μl), mixed, and then centrifuged for 10 min at 5400 ×g. Brains were homogenized in 800 μl of BSA-LR with a bead beater for 30 sec at 4800 rpm twice. Following centrifugation at 5400 ×g for 15 min, a portion of the resulting supernatant was added to an equal volume of 30% TCA, mixed, and then centrifuged at 5400 ×g for 10 min. The radioactivity in the resulting supernatant and pellet (Sup and Pel fractions) for serum and brain was calculated separately, and the percent of radioactivity in the precipitate was calculated using the following equation:

(1) $$\%\;\;\mathrm{Acid}\;\;\mathrm{Precip}=100\times \left(\mathrm{Pel}\right)/\left(\mathrm{Sup}+\mathrm{Pel}\right)$$

To correct for degradation that might have occurred during the processing, ^125^I/^14^C-IRA was added to non-radioactive arterial whole blood or to whole brain and processed as above. The biological samples were corrected for degradation during processing by dividing their values by those for processing control (Pcon) values and multiplying by 100. This can sometimes result in a value greater than 100%. Pcon values are listed in Table 2.

**Table 2.** In vivo degradation of ^125^I/^14^C-IRA peptides within 1 hour.

Peptide	Whole Brain			Serum		
	10 min	60 min	Pcon	10 min	60 min	Pcon
*Single IRAs:*						
Albiglutide (BAF)	43.35 ± 2.9	40.9 ± 9.3	66.6 ± 0.3	53.96 ± 0.4	20.71 ± 1.3^a^	94.3 ± 0.4
Dulaglutide (BAF)	58.42 ± 0.3	37.47 ± 4.1	83.5 ± 1.6	84.67 ± 0.8	39.1 ± 1.4^a^	97.1 ± 0.1
*Dual IRAs:*						
DA5-CH	79.7 ± 12.9	77.61 ± 22.3	89.6 ± 0.2	83.83 ± 4.9	49.35 ± 2.5^a^	75.3 ± 1.1
Tirzepatide	100.7 ± 0.0	99.73 ± 0.6	99.1 ± 0.2	100.5 ± 0.0	99.65 ± 0.1	99.3 ± 0.0

*The values shown are mean ± SEM percentages of ^125^I/^14^C-labeled IRAs injected iv that remained intact 10 or 60 min later as reflected in accumulation of radioactive degradation products with time as determined by precipitation of those products. Data are expressed relative to processing controls (Pcon, *n* = 2) with *n* = 3 per group.

^a^*p* < 0.05 vs values at 10 min post injection.

### 2.6. Clearance of ^125^I*/*^14^C-IRAs from serum

**Figure 1.**
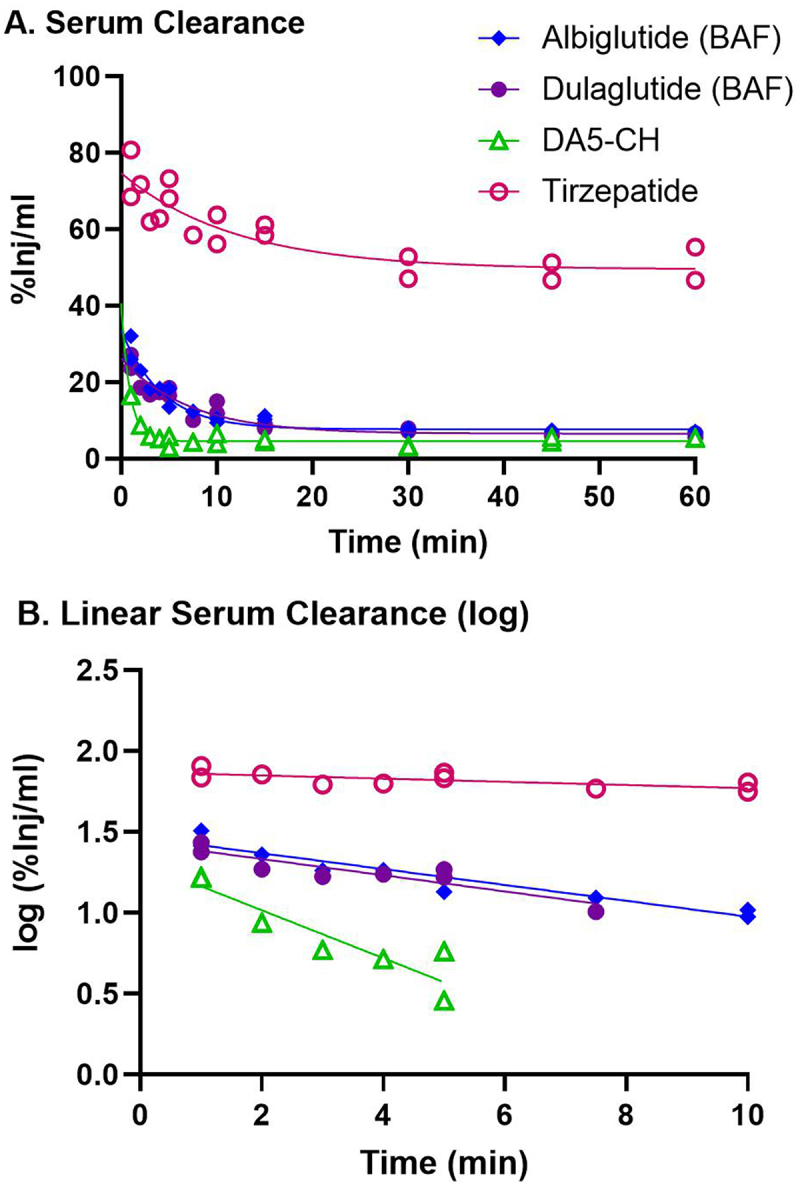
Clearance of ^125^I/^14^C-IRAs from serum.

Following anesthetization, the jugular vein and right carotid artery were exposed. Mice were given a jugular vein injection of 0.2 ml BSA-LR containing 3 × 10^5^ cpm-1×10^6^ cpm of an ^125^I/^14^C-labeled IRA and 5 × 10^5^ cpm of ^99m^Tc-Alb. Blood samples were collected from the carotid artery at various time points from 1 to 60 min after iv injection (see Figure 1). Mice were then decapitated, and their brains were removed and weighed. After clotting, the arterial blood was centrifuged at 3200 ×g for 10 min. Levels of radioactivity in serum (50 μl) and brain were measured in a gamma counter for ^125^I and ^99m^Tc for 3 min. After ^99m^Tc decay, ^14^C-DA5-CH samples were solubilized with Solvable (Sigma), transferred to scintillation vials containing Ecoscint (National Diagnostics) and ^14^C was measured in a beta counter. Sample injection checks were taken throughout the course of the study to calculate the mean injected cpm/mouse. To determine the rate of ^125^I/^14^C-IRA clearance from the serum, the results were expressed as the percentage of an injected dose in each ml of serum (%Inj/ml), which was calculated by dividing the cpm in a ml of serum (cpm/ml serum) by the cpm injected into the mouse (cpm/Inj):

a) The level of radioactivity (%Inj/ml) in serum over the 60 min time frame. b) The linear distribution phase (log(%Inj/ml)) is used to calculate the clearance. The half-time clearance from blood for each IRA is listed in Table 3. *n* = 18 mice per IRA.

**Table 3.** Pharmacokinetics of IRA peptide brain uptake within 1 hour.

	Half-Time	Blood to Brain Influx			Level of Brain	
	Serum	Unidirectional			Vascular	
	Clearance	Infux Rate			Binding	Percent in Parenchyma
Peptide	(t_1/2_)	(*K*_i_, µl/g-min)	r with time	p	(*V*_r_, µl/g)	vs. Capillaries
*Single IRAs:*						
Albiglutide (BAF)	6.14	1.379 ± 0.086	0.9809	<0.0001	2.899 ± 1.15	89.4
Dulaglutide (BAF)	6.02	1.095 ± 0.072	0.9789	<0.0001	−0.147 ± 0.89	61.8
*Dual IRAs:*						
DA5-CH (Model 1)	2.03	0.242 ± 0.128	0.48	0.0831	11.20 ± 3.05	57.6
DA5-CH (Model 2)	”	2.476 ± 0.533	–	0.0433	0	”
Tirzepatide	30.62	NS^a^	0.04	0.8807	0.03 ± 0.15	N/A^b^

^a^NS = not significant. This reflects the observation that brain to serum ratios of an iv injected labeled peptide never differed significantly from baseline during the 60 minute circulation period. Even in these cases, it is still possible to determine the correlation (r) between the B/S ratios of the labeled peptide in relation to time.

^b^N/A = not applicable. For those IRAs that did not have a significant rate of transport, we are not reporting the amount present in the parenchyma vs. capillaries. A dash (–) symbol indicates the value could not be calculated for this data and quotes (”) indicate the same value as above.

(2) $$\%\mathrm{Inj}/\mathrm{ml}=100\times \left(\mathrm{cpm}/\mathrm{mlserum}\right)/\left(\mathrm{meancpm}/\mathrm{Inj}\right)$$

The log(%Inj/ml) was plotted against time to calculate the rate of clearance from the serum. To calculate the half-time clearance in minutes, the inverse slope (*K*_i_) of the curve for each IRA was multiplied by 0.301 (log_10_^2^). The blood and brain cpm data were used for the measurement of brain influx and initial volume of distribution in brain as described in Section 2.7.

### 2.7. Measurement of brain influx and initial volume of distribution in brain

To calculate the blood-to-brain unidirectional influx constant (*K*_i_), multiple-time regression analysis was used as first described by Patlak, Blasberg, and Fenstermacher.^67,68^ The brain/serum (B/S) ratios (μl/g) of the ^125^I/^14^C-IRA in each gram of brain were calculated from the data in Section 2.6 and plotted against their respective exposure times as previously applied by Rhea et al. 2018.^69^ Exposure time differs from clock time in that exposure time corrects for clearance of the ^125^I/^14^C-IRA from blood and thus, better represents the amount of ^125^I/^14^C-IRA available for BBB transport at any given time. Delta B/S ratios were calculated by subtracting the vascular space as measured by the B/S ratio for ^99m^Tc-Alb from the B/S ratio for the respective ^125^I/^14^C-IRA. Thus, delta B/S ratios represent material that has been taken up by or has crossed the BBB. The slope of the linear portion of the relation between delta B/S ratios and exposure time defines *K*_i_ (μl/g-min) and is reported with its error term. This represents Model 1 for DA5-CH. The y-intercept of this linear portion of the relation defines *V*_i_, (μl/g), the initial volume of distribution in brain at *t* = 0, which is the functional volume per unit brain mass of a soluble compound that exchanges rapidly and reversibly with plasma.^68^ *V*_i_ consists of the vascular space (*V*_v_) and any residual space after accounting for *V*_v_ and usually represents vascular binding (*V*_r_). Since we correct each ^125^I/^14^C-IRA delta B/S for vascular space, our y-intercept values likely represent vascular binding. For DA5-CH, there appeared to be rapid, early transport that would indicate a *V*_r_ equal to 0. Therefore, using *V*_r_ equal to 0, we calculated an estimate of this new *K*_i_ and refer to it throughout the manuscript as Model 2.

### 2.8. Percent of injected dose taken up by the brain

We also expressed the results for the data as the percentage of the iv injected dose taken up by each gram of brain tissue (%Inj/g) for those IRAs that showed transport into the brain:

(3) $$\%\mathrm{Inj}/g=(\mathrm{delta}\;\;B/S-V_{r})x\left(\%\mathrm{Inj}/\mathrm{ml}\right)$$

Where delta B/S is the vascular corrected B/S value for each IRA, and *V*_r_ corrects the B/S ratio for the amount of peptide reversibly binding the endothelium. This value is then multiplied by the %Inj/ml of each ^125^I/^14^C-IRA at each time point. After plotting the %Inj/g at each time point for each IRA, we calculated the area under the curve (AUC) to better identify the amount of brain exposure to each IRA over the entire time course.

### 2.9. Saturability of brain uptake

We then identified whether brain uptake of each IRA had a saturable component. Anesthetized mice were co-injected iv with 3 × 10^5^ cpm-1×10^6^ cpm ^125^I/^14^C-IRA and 5 × 10^5^ cpm of ^99m^Tc-Alb in 0.2 ml BSA-LR with or without 1 μg of the non-radioactive IRA. Fifteen minutes after iv injection, blood was collected from the right carotid artery, and the whole brain was removed and weighed. Samples were counted for radioactivity. Results were expressed as delta B/S ratios in units of μl/g.

### 2.10. Capillary depletion without vascular washout

The capillary depletion method was used to separate cerebral capillaries from brain parenchyma. Anesthetized mice received an iv injection of 3 × 10^5^-1×10^6^ cpm of an ^125^I-IRA with 5 × 10^5^ cpm ^99m^Tc-Alb BSA-LR and 15 min later, blood and brains were collected. Whole brains were homogenized with physiological buffer (10 mM HEPES, 141 mM NaCl, 4 mM KCl, 2.8 mM CaCl_2_, 1 mM MgSO_4_, 1 mM NaH_2_PO_4_, and 10 mM D-glucose adjusted to pH 7.4) using two different sized glass dounce homogenizers, mixed with 26% dextran (Sigma-Aldrich), and centrifuged at 5400 ×g for 15 min at 4°C.^69^ The pellet, containing the capillaries, and the supernatant, containing the brain parenchymal/interstitial fluid space, were carefully separated. The ratio of ^125^I-IRA radioactivity in the supernatant (parenchyma) was corrected for vascular space by subtracting the B/S ratio of ^99m^Tc-Alb in the supernatant. The corrected parenchyma/serum and capillary/serum ratios (μl/g) were calculated as (cpm/g tissue)/(cpm/μl) serum.

### 2.11. Long-term measurement of brain uptake: liraglutide, semaglutide, tirzepatide

Substances that are enzymatically stable in blood and have slow clearance from the circulation can slowly enter the brain using the extracellular pathways.^63,64^ As some of the IRAs which we found did not cross the BBB in our 60 min studies exhibit these characteristics, we considered whether these IRAs would be taken up by the brain following a longer study period of 6 h. Albumin enters the brain by extracellular pathways,^70^ and so we used albumin in two ways: we used ^99m^Tc-Alb circulating for 10 min to measure vascular space and ^99m^Tc-Alb circulating for 6 h to measure brain uptake by extracellular pathways. This is similar to the time-differential methodology developed by Poduslo and Curran to measure a substance’s vascular space and its uptake rate.^71^ Following anesthetization, the jugular vein and right carotid artery were exposed. Mice were given an iv injection of 0.1% BSA-LR containing 1 × 10^6^ cpm of ^125^I-IRA or 5 × 10^5^ cpm of ^99m^Tc-Alb, which circulated for 6 h. Mice receiving only the ^125^I-IRA were given an injection of 5 × 10^5^ cpm of ^99m^Tc-Alb 10 min prior to the end of the 6 h circulation time to act as a vascular marker. A separate set of mice received an iv injection of both 1 × 10^6^ cpm of ^125^I-IRA and 5 × 10^5^ cpm of ^99m^Tc-Alb that circulated for 10 min. Following the 10 min or 6 h circulation time points, blood and brain were collected as described above. Thus, we had B/S ratios for ^99m^Tc-Alb measuring vascular space (10 min circulation time) and B/S ratios for ^99m^Tc-Alb measuring vascular space plus uptake by the extracellular pathways (6 h circulation time) which could be compared to the B/S ratios for the ^125^I-IRAs after 10 min and 6 h of circulation.

Immediately after counting the radioactivity, the blood and brains from the 10 min and 6 h time points were processed as described in Section 2.5 for in vivo stability. For further comparison, data from the 1 h time point were taken from the original pharmacokinetic uptake study. The ^125^I-IRA brain % acid precipitation (%AP) was divided by the ^125^I-IRA serum %AP at each time point to calculate the %AP B/S ratio. This value was multiplied by the B/S ratio for each ^125^I-IRA to correct for degradation over the 10 min, 1 h, or 6 h time periods. Corrected AP B/S ratios were then corrected for vascular space by subtracting the ^99m^Tc-Alb B/S ratio within each mouse for the 10 min and 6 h time points (AP corrected delta B/S ratio). For the 1 h time point, the average ^99m^Tc-Alb B/S ratio (10.03 μl/g) at 10 min across all ^125^I-IRAs was used for correction. The AP corrected delta B/S ratio for each ^125^I-IRA was then multiplied by the %Inj/ml within each mouse to calculate the AP corrected delta %Inj/g.

To estimate the *K*_i_ for each of these three ^125^I-IRAs, we included their 6 h delta B/S ratios with their 1 h delta B/S ratios in the linear regression model. The entry rate for ^99m^Tc-Alb was calculated by using the ^99m^Tc-Alb 1 h pharmacokinetic data from ^125^I-tirzepatide and the 6 h time point for ^99m^Tc-Alb. For the 1 h and 6 h ^99m^Tc-Alb B/S ratios, we calculated their delta B/S values by subtracting the 10 min B/S ratio for ^99m^Tc-Alb (10.03 μl/g). The *K*_i_ for each of these IRAs and for albumin was calculated as described in Section 2.7 using the 1–60 min and 6 h values.

### 2.12. Statistical analyses

Regression analysis, area under the curve (AUC), and other statistical analyses were performed with the use of Prism 9.0 (GraphPad Software Inc., San Diego, CA). Means are reported with their standard errors and compared by one-way analysis of variance (ANOVA) followed by the Newman–Keuls post-hoc test. Linear and non-linear regression lines are reported with their correlation coefficients (r) and *n* values. Multiple-time regression analyses were used to compare changes in variables over time. Statistical significance was defined as p < 0.05.

## 3. Results

### 3.1. Properties of the IRAs studied

Table 1 provides basic information on the IRAs studied in this report. Two are single IRAs (albiglutide (BAF) and dulaglutide (BAF)). The other two are dual IRAs (DA5-CH and tirzepatide). DA5-CH is a variant of peptide 18 of Finan and Ma et al. (2013) with an 11 amino acid N-terminal extension (see Table 1). The molecular weights of the IRAs all less than 5.6 kDa. Albiglutide (BAF) and DA5-CH are positively charged, while dulaglutide (BAF) and tirzepatide are negatively charged. The newest and only dual IRA approved by the FDA, tirzepatide (Mounjaro) proved significantly more lipid soluble than the other tested IRAs here (partition coefficient = 0.132 ± 0.01) but was still less than that of another acylated IRA we previously tested, peptide 19 (partition coefficient = 0.249).^50^ That peptide was mistakenly labeled as peptide 18 in the report just cited as explained in the footnote to the Introduction.

### 3.2. Degradation in serum and whole brain

To identify whether the radioactively labeled IRAs were stable in blood and brain, we acid precipitated the peptides and measured the radioactivity obtained in serum and brain homogenates. Table 2 shows the amount of ^125^I/^14^C precipitated with acid at 10 and 60 min after correction for processing. The majority of all radioactive IRAs were intact in serum at 10 min, when the blood-to-brain unidirectional influx rates are calculated (Section 3.4). By 60 min, there was an increase in the amount of IRA degraded in serum, except for ^125^I-tirzepatide. There were no significant differences between 10 min and 60 min in degradation in whole brain.

### 3.3. Clearance from serum

Figure 1a shows the clearance of ^125^I/^14^C-IRAs from serum up to 1 h after iv injection. The relation between log levels of the radioactivity in arterial serum expressed as a percentage of injected IRA per ml (%Inj/ml) over time during the linear period after iv injection is shown in Figure 1b. Linear regression analysis showed a statistically significant relation between log (%Inj/ml) and time for all peptides. For this early time period,^14^C-DA5-CH had the fastest early-phase clearance (2.03 min), while ^125^I-tirzepatide had the slowest clearance (30.6 min). The early period half-time clearance rates for all the peptides tested are listed in Table 3.

### 3.4. Whole brain influx and initial volume of distribution

**Figure 2.**
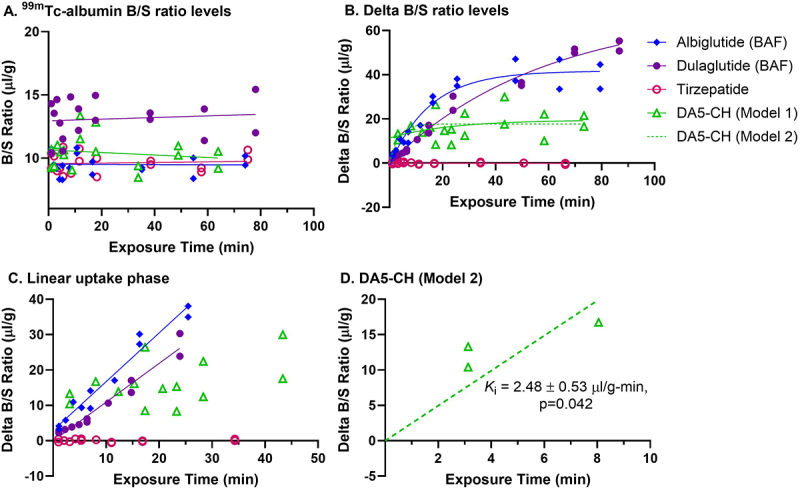
BBB pharmacokinetics within one hour of ^125^I/^14^C-IRAs and ^99m^Tc-albumin.

The brain influx rate (*K*_i_) for the four ^125^I/^14^C-IRAs investigated here was calculated from the delta B/S ratios and is graphed in Figure 2. As expected, there was no significant brain uptake of the vascular marker, ^99m^Tc-Alb, within the 1 h time period (Figure 2a). The full delta B/S ratio curve over the course of 1 h is presented in Figure 2b, while the linear phase of transport is shown in Figure 2c. The linear phase of transport is used to calculate the unidirectional influx rate. A blood-to-brain unidirectional influx rate (*K*_i_) was measurable for ^125^I-albiglutide (BAF) and ^125^I-dulaglutide (BAF) (Table 3). There was no significant (ns) relationship between the delta B/S ratios and exposure time for ^125^I-tirzepatide. There was a trend (*p* < 0.1) toward a relationship between the delta B/S ratios and exposure time for ^14^C-DA5-CH. The lack of a significant linear relation with time led us to conclude that ^14^C-DA5-CH did not cross the BBB, which we term Model 1 for this compound. However, the delta B/S values were greater than zero and upon closer investigation, there appeared to be a rapid, early uptake phase for ^14^C-DA5-CH that quickly comes to equilibrium. When *V*_r_ is set to 0, which we term Model 2 for this compound, *K*_i_ = 2.48 ± 0.53 μl/g – min (*p* = 0.043; Figure 2d). Additional studies are needed to definitively establish *V*_r_ for DA5-CH and hence its *K*_i_. *K*_i_ and *V*_r_ values for both Model 1 and Model 2 of DA5-CH are thus listed in Table 3, and their delta B/S data are graphed in Figure 2.

**Figure 3.**
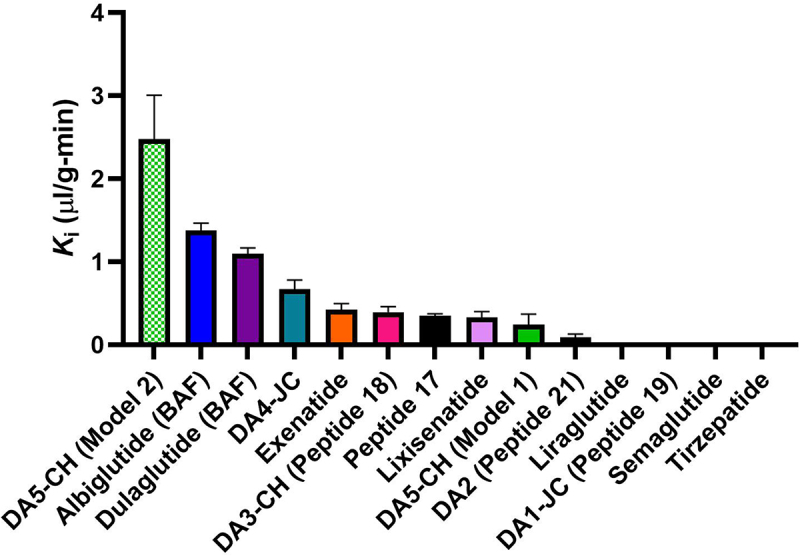
Rate constants (*K*_i_) of ^125^I/^14^C-IRAs transport into whole brain within one hour.

a) Vascular space represents brain-to-serum ratio (B/S) of ^99m^Tc-albumin. b) Delta B/S ratios of ^125^I/^14^C-IRAs, corrected for vascular space, present in whole brain over the entire time curve. *n* = 18 mice per IRA. c) The linear phase of transport present in (b) is used to calculate the unidirectional influx rate, *K*_i_, presented in Figure 3 and Table 3. Tirzepatide and DA5-CH Model 1 (ns) did not significantly differ from 0. d) Early time points for DA5-CH suggest early uptake into brain (Model 2).

The unidirectional blood-to-brain influx rate (*K*_i_) calculated from Figure 2c for all four IRAs tested is graphed with previously calculated *K*_i_’s for other IRAs^50^ in Figure 3 by rank. The rates of transport are statistically different among the IRAs (*p* < 0.001). ^125^I-Albiglutide (BAF) has one of the fastest *K*_i_ (1.38 ± 0.09 μl/g – min, *r* = 0.98, *p* < 0.0001). This rate is over two-fold faster than the fastest IRA previously tested, which was for ^125^I-DA4-JC (*K*_i_ = 0.668 ± 0.12 μl/g – min).^50 14^C-DA5-CH Model 2 is likely an overestimate of the *K*_i_ for this IRA as the model eliminates all vascular binding, setting *V*_r_ = 0. The true *K*_i_ for DA5-CH is likely somewhere between 0.242 ± 0.128 μl/g and 2.476 ± 0.533 μl/g.

The unidirectional influx rates, *K*_i_ (slope) and *V*_r_ (y-intercept), are listed in Table 3 or in our previous report.^50^ *n* = 11–14 per IRA. DA peptides are experimental dual IRA agonists created by Christian Hölscher.^38,62^ Peptides 18, 19, and 21 are dual IRAs created by Finan and Ma et al. (2013).^72^ numbered as in their Supplementary Fig. S1.

In the current study, the level of brain vascular binding (*V*_r_) at *t* = 0 min was highest for ^14^C-DA5-CH Model 1 at 11.20 ± 3.1 μl/g. This is likely an overestimation of Vr as it is probable that DA5-CH has as one of its kinetic components a rapid influx and comes quickly to equilibrium. This would result in a *V*_r_ that is somewhere between 0 and 11.2 μl/g. The *V*_r_ for ^125^I-dulaglutide (BAF) was the lowest at −0.15 μl/g ±0.9. *V*_r_‘s for the other IRAs are listed in Table 3.

**Figure 4.**
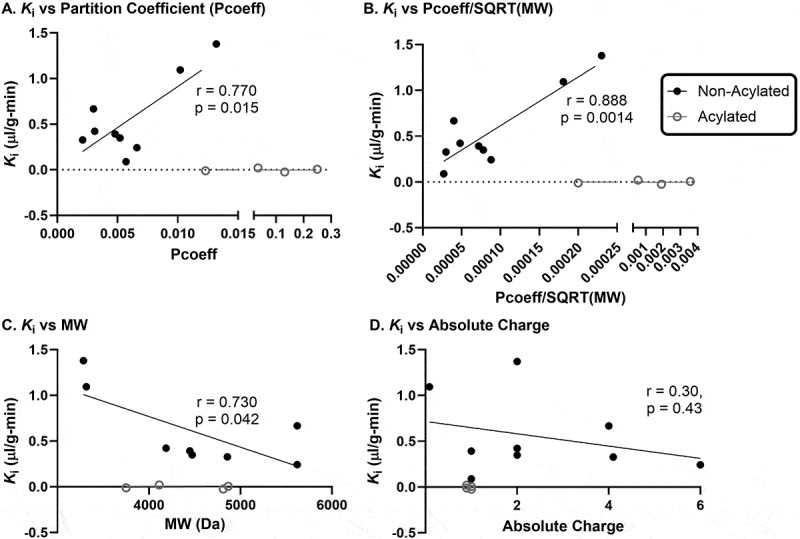
Correlations between the unidirectional influx rate (*K*_i_) and characteristics of each IRA (Table 1 and previously reported (Salameh et al., 2020)).

In order to investigate the relation between the *K*_i_ values with the characteristics of each IRA (solubility, MW, charge), we combined the data from the IRAs reported here and previously.^50^ (Figure 4). For these correlations, we used the *K*_i_ calculated from the standard criterion for DA5-CH (Model 1). Acylation improves lipid solubility and noticing that 4 of the IRAs with low *K*_i_’s are acylated, we separated these IRAs from our overall correlations. The *K*_i_ did significantly correlated with the solubility octanol/buffer partition coefficient (Figure 4a) or the solubility octanol/buffer partition coefficient divided by the square root of the molecular weight (Figure 4b), and. co molecular weight (Figure 4c). There was no correlation between the *K*_i_ and the absolute net charge (*p*= 0.43, Figure 4d).

Characteristics include the a) solubility octanol partition coefficient (Pcoeff), b) Pcoeff divided by the square root of the molecular weight (Pcoeff/SQRT (MW)), c) log(Pcoeff/SQRT (MW)), and d) absolute charge. Pearson correlation *r* value is presented with the *p* value. DA5-CH (Model 1) *K*_i_ was used.

**Figure 5.**
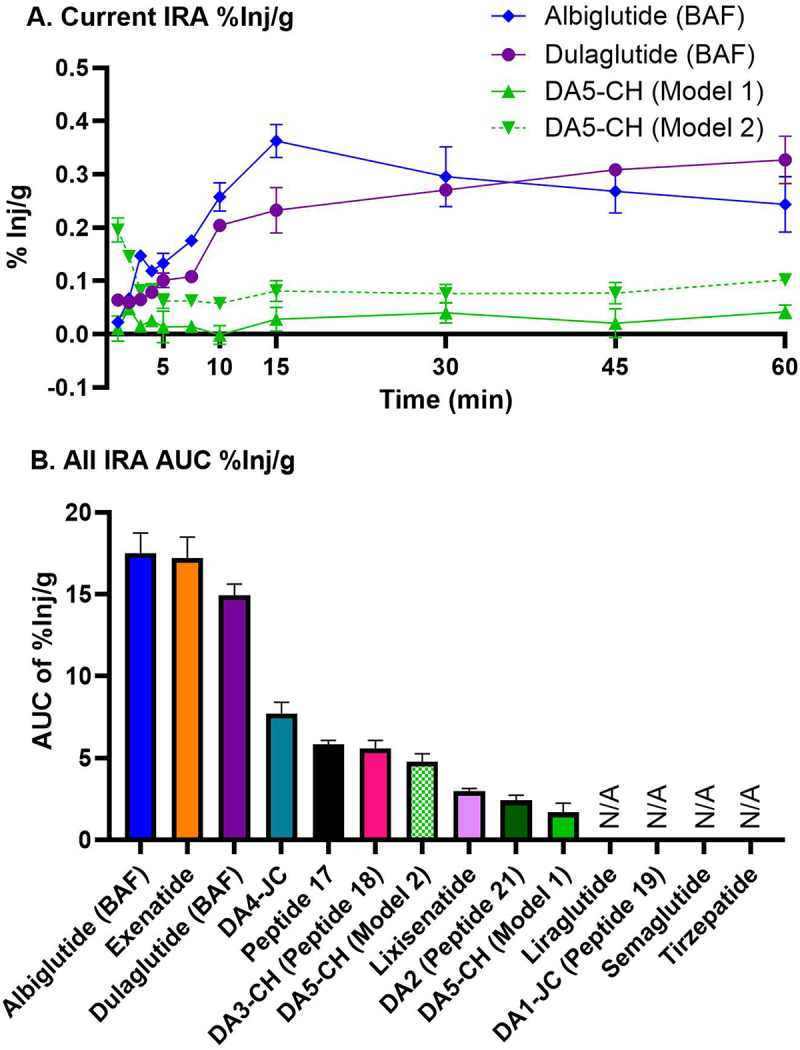
Percent of the iv injected dose of ^125^I/^14^C-IRAs taken up per gram of brain tissue (%Inj/g).

The percent of injected ^125^I/^14^C-IRAs taken up per gram brain tissue (%Inj/g) is shown in Figure 5a except for ^125^I-tirzepatide that did not exhibit significant BBB transport. The data are based on the delta B/S ratios depicted in Figure 2b and are corrected for the *V*_r_. DA5-CH (Model 2) assumes there is no vascular binding. By 15 min, the percentage was highest for ^125^I-albiglutide (BAF) (0.36%Inj/g). At 60 min, the %Inj/g was 0.33% and continuing to rise for ^125^I-dulaglutide (BAF). We also calculated the AUC of the %Inj/g from the current IRAs and our previously published study (see Figure 4 in^50^ and Figure 5b here). Albiglutide, exenatide, and dulaglutide (BAF) all had a %Inj/g AUC greater than or equal to 15, as expected based on the %Inj/g graph. Lixisenatide, peptide 21, and DA5-CH (model 1) had lower levels with less than a %Inj/g AUC of 3. These values represent a nearly sixfold range in distribution for brain uptake of these IRAs.

a) Current IRA %Inj/g corrected for the initial level of vascular binding (*V*_r_), except for DA5-CH Model 2 which uses *V*_r_ = 0; *n* = 1–2/time point per IRA. Tirzepatide was not included as there was no measurable transport. b) The area under the curve (AUC) (from current and previous publication.^50^) was calculated and presented in rank order; *n* = 18–22 per IRA. N/A= not applicable as %Inj/g is not calculated from those that did not have significant transport within the 1 hour. DA peptides are experimental dual IRA agonists created by Christian Hölscher.^38,62^ Peptides 18, 19, and 21 are dual IRAs created by Finan and Ma et al. (2013).^72^ as numbered in their Supplementary Fig. S1.

### 3.5. Saturability of transport across the BBB

To determine if transport of each IRA occurred in a saturable manner, we measured IRA whole-brain uptake after iv injection of each ^125^I/^14^C-IRA with or without its non-radioactive form. Inclusion of the non-radioactive IRA (1 μg/mouse) with the ^125^I/^14^C -IRA 15 min after injection did not affect the delta B/S ratios of any ^125^I/^14^C-IRA tested (Table 4). Serum %Inj/ml was decreased when ^125^I-dulaglutide (BAF) was co-injected with unlabeled dulaglutide (BAF) (11.18 ± 0.67%Inj/ml vs. 7.45 ± 0.31%Inj/ml, *p* < 0.05).

**Table 4.** Saturability of IRA peptide transport into brain.

Peptide	Whole Brain		Serum	
	Without unlabeled IRA	With unlabeled IRA	Without unlabeled IRA	With unlabeled IRA
*Single IRAs:*				
Albiglutide (BAF)	39.3 ± 1.50	37.82 ± 1.76	9.11 ± 0.43	8.47 ± 0.40
Dulaglutide (BAF)	17.86 ± 0.99	19.77 ± 1.36	11.18 ± 0.67	7.45 ± 0.31^a^
*Dual IRAs:*				
DA5-CH	26.25 ± 8.07	18.7 ± 4.58	5.27 ± 0.71	6.60 ± 0.63
Tirzepatide	−0.16 ± 0.13	0.10 ± 0.13	58.47 ± 5.68	55.74 ± 1.37

The values shown are mean ± SEM of delta brain/serum ratios (μl/g) at 15 min for whole brain and %Inj/ml for serum (*n* = 7–8 per group). Data were analyzed by a two-tailed, unpaired t test; ^a^ *p* < 0.05 comparing samples with or without unlabeled IRA peptides.

### 3.6. Uptake by brain parenchyma

To determine if the ^125^I/^14^C-IRAs were transported into brain parenchyma rather than being sequestered by vascular endothelial cells, we performed capillary depletion studies on those IRAs that exhibited blood-to-brain transport. This method distinguishes between peptide that has infiltrated brain parenchyma from that which is sequestered by brain endothelial cells. All IRAs exhibiting a significant rate of transport into the brain were predominantly present in the parenchyma compared to the capillaries (Table 3). The percent of each IRA reaching the brain that was found in parenchyma vs capillaries (Table 3) was highest for albiglutide (BAF) (89.4%) followed by dulaglutide (BAF) (61.8%) and then DA5-CH (57.6%). This further supports entry of DA5-CH into the brain.

### 3.7. Re-evaluating serum-stable IRAs for brain uptake after prolonged (6 h) circulation

**Figure 6.**
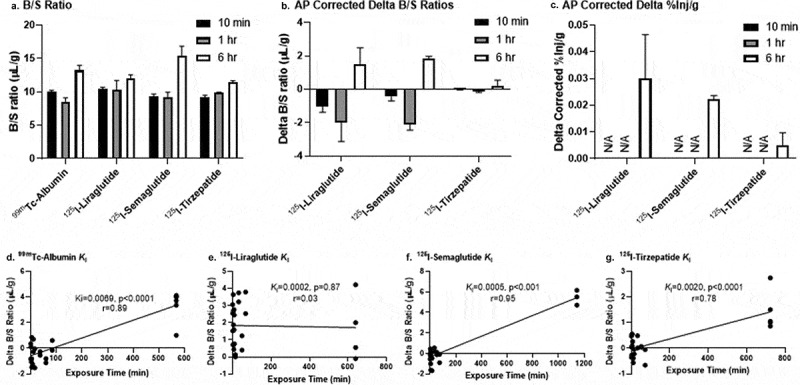
^125^I-IRA and ^99m^Tc-albumin brain entry over prolonged period of time.

Many of the long-lasting IRAs (liraglutide, semaglutide, tirzepatide) we injected here and in an earlier study^50^ remained intact in serum 1 h later, potentially allowing uptake at later times. This raised the possibility that IRAs undetectable in the brain at the 1 h time point might reflect very slow uptake and that detection might occur with a longer circulation time. We tested this on three ^125^I-labeled IRAs not detectable in the brain at 1 h, by allowing a 6 h interval between drug injection and collection of serum and brain tissue. The 6 h interval was appropriate given that mouse brain levels of iv administered semaglutide administered 6 h earlier are very similar to steady state levels after 5 d of daily dosing (see,^73^ Figs. 2a-b). These experiments were run on labeled liraglutide, semaglutide, and tirzepatide, the reported half-lives of which in human serum are long, specifically 11–13 h, ~160 h, and 111–124 h, respectively.^54,60^ The majority (>50% AP) of each labeled IRA was still intact in serum at 6 h (Table 5). Substances with long circulating half-lives, small volumes of distribution, and resistance to enzymatic degradation may be able to enter the brain by extracellular pathways.^64,70^ Serum clearance continued to occur for all IRAs at 6 h as expected (data not shown). All these IRAs had greater B/S ratios at 6 h compared to 10 min (Figure 6a), the latter time selected for being within the linear uptake phase in the 1 h pharmacokinetic study. We detected roughly equivalent amounts of liraglutide and semaglutide present in brain when corrected for vascular contribution and degradation, with very little tirzepatide present (Figure 6b). For liraglutide, semaglutide, and tirzepatide, the calculated amount present in brain after correction for the amount present in blood was estimated to be 0.03, 0.022, and 0.005%Inj/g, respectively (Figure 6c). If we approximate the *K*_i_ for each IRA using time points at 10 min, 1 h, and 6 h, the amount of albumin entering the brain by way of the extracellular pathways exceeds that for these three IRAs (Figure 6d-g). These results suggest that longer-lasting IRAs such as semaglutide and tirzepatide can enter the brain slowly and in relatively small amounts through the extracellular pathways, while liraglutide appears to remain adhered to or sequestered by the brain endothelial cells comprising the BBB.

a) B/S ratios of ^125^I-IRA and ^99m^Tc-albumin at 10 min, 1 hr, and 6 hr. ^99m^Tc-albumin levels were pooled from three separate cohorts of mice at 10 min (n = 18) and 1 hr (n = 6). ^99m^Tc-albumin levels at 6 hr are from an independent study (n = 4). ^125^I-IRA values at 10 min and 6 hr represent n = 3–6. ^125^I-IRA values at 1 hr are from the 1 hr multiple-time regression study in Figure 2 and previously published.^50^ b) AP Corrected Delta B/S ratios for each IRA are calculated by multiplying the B/S ratio in panel A by the B/S AP% degradation ratio at each time point (see Table 5 for 10 min and 6 hr and Table 2 for 1 hr [Tirz] and Table 2 in Salameh.^50^ for 1 hr [Lira, Sema]). This AP corrected B/S ratio was then subtracted by the respective ^99m^Tc-albumin B/S ratio that circulated for 10 min within each mouse, except for the 1 hr data (the 1 hr ^125^I-IRA B/S ratio subtracted the mean value reported in panel A for ^99m^Tc-albumin at 10 min (10.03 μL/g), n = 2/IRA- so not within mouse). c) Following correction by AP, the %Inj/g of each ^125^I-IRA taken up by brain is represented. B/S ratios presented in panel A were multiplied by the B/S ratio for the AP value at each time point, ^99m^Tc-albumin and then multiplied by the respective %Inj/ml value to calculate the AP corrected %Inj/g (n = 3–6). N/A = not applicable as a negative Delta B/S ratio would not result in positive %Inj/g values. d-g) *K*_i_’s for albumin and the IRAs were re-calculated based on the delta B/S ratios at 1–60 min and 6 h clock time. The *K*_i_, *p* value, and correlation coefficient (r) for each substrate’s multiple-time regression analysis are shown in each panel.

**Table 5.** In vivo degradation of 125I-IRA peptides at 6 hours.

Peptide	Whole Brain			Serum		
	10 min	6 hr	P_con_	10 min	6 hr	P_con_
*Single IRAs:*						
Liraglutide	94.8 ± 1.47	104.5 ± 9.51	82.9	107.6 ± 0.17	105.7 ± 0.49	90.7
Semaglutide	102.6 ± 2.03	65.7 ± 5.45^a^	99.1	100.5 ± 0.01	85.6 ± 1.39^a^	99.7
*Dual IRA:*						
Tirzepatide	99.6 ± 0.81	85.9 ± 2.59	99.1	100.5 ± 0.03	96.0 ± 0.55^a^	99.3

The values shown are mean ± SEM percentages of ^125^I-labeled IRAs injected iv that remained intact 10 min or 6 hr later as reflected in accumulation of radioactive degradation products with time as determined by precipitation of those products. Data are expressed relative to processing controls (P_con_, *n* = 1–2) with *n* = 3 per group. Data were analyzed by a two-tailed, unpaired t test; ^a^ *p* < 0.05 comparing 10 min to 6 hr.

## 4. Discussion

Evaluating the relative therapeutic potential of systemically administered drugs showing promise in neurodegenerative disease such as AD and PD requires understanding their BBB transport pharmacokinetics. The present study extends our previously published work^50^ on BBB transport characteristics of nine IRAs that show promise as AD and PD therapeutics. Here, we provide the same pharmacokinetic data on brain uptake of four additional IRAs: the GLP-1 R single IRAs albiglutide (BAF) and dulaglutide (BAF) and the dual GLP-1 R/GIPR dual IRAs DA5-CH and tirzepatide. In addition, we have investigated whether long-lasting IRAs can enter the CNS over a longer period of time.

### 4.1. Stability of ^125^I-IRAs in serum and whole brain

At 10 min, the majority of radioactivity was recovered in precipitated peptides for each radioactively labeled IRA. This time point is within the linear brain uptake phase during which we calculate the transport rate, suggesting that the majority of peptide transported is intact at 10 min. There are many explanations for the degradation we did observe with some of the IRAs by 60 min. First, the ^125^I radioactive tag could be cleaved off these peptides by iodinases, which would still allow for an intact, but unlabeled, IRA. Second, the IRA peptides themselves could be degraded into multiple fragments, which could still be bioactive. Third, there could be species differences in the clearances and degradations of the IRAs.^74^ IRAs that reached the brain (albiglutide (BAF) and dulaglutide (BAF)) remained stable for 1 h, indicating that there was no significant decrease in stability of these IRAs in the brain from 10 min to 1 h.

### 4.2. Relative brain influx rates (K*_i_*’s) of IRAs and their saturability

Albiglutide (BAF) and dulaglutide (BAF) had the fastest influx rates in this study. Of all the peptides studied, ^14^C-DA5-CH had the most problematic pharmacokinetic profile. Its delta B/S ratio does not significantly correlate with exposure time (Model 1). This is usually interpreted as meaning there is no blood-to-brain transfer. The delta B/S ratios, however, are clearly above zero (unlike the case for tirzepatide), indicating an uptake space in addition to the vascular space. Sequestration by brain endothelial cells was considered a possible explanation of DA5-CH’s significant extravascular distribution in the absence of significant change in B/S ratios over time. Finding a greater percentage of DA5-CH present in the brain parenchyma further supports entry of DA5-CH into the brain. Consequently, the lack of a correlation between delta B/S ratios and time for ^14^C-DA5-CH probably reflects a rapid uptake phase followed by an early equilibrium phase (Model 2, Figure 2d). Because there were few early time points in the DA5-CH curve, it is difficult to accurately measure the *K*_i_ of this IRA, but it is probably between 0.24 and 2.5 µl/g-min.

For the nine IRAs we have studied that had a significantly measurable uptake rate at 60 min of study, the relative rates (*K*_i_) of significant IRA brain uptake 1 h after iv injection were as follows in rank order: DA5-CH, Model 2 (*K*_i_ = 2.476) > albiglutide (BAF) (1.379) > dulaglutide (BAF) (1.095) >> DA4-JC (0.6680) > exenatide (0.4231) > DA3-CH/Peptide 18 (0.3922) > Peptide 17 (0.3489) > lixisenatide (0.3271) > DA5-CH, Model 1 (0.242) ≫ DA2/Peptide 21 (0.0892). This indicates a greater than 15-fold range in brain entry rates of IRAs within 1 h of iv injection, highlighting the great variability in BBB transport rates in this family of agonists.

Upon correlating the *K*_i_ values with physical properties of the peptide IRAs (lipid solubility, molecular weight, and charge), we identified that lipid solubility and molecular weight are the best predictors of *K*_i_. The more lipophilic, the faster the rate of transport. Therefore, transcellular diffusion as mediated by lipid solubility and molecular weight seems to be a predominant mechanism for transport of nonacylated peptide IRAs. The inability of the absolute charge model to predict BBB permeability suggests that charge interactions may not be the main mechanism by which these substances cross the BBB as we had previously suggested. However, as we have previously shown that enhancement of adsorptive transcytosis with wheatgerm agglutinin which acts via charge increases transport of exenatide,^50^ it is still possible adsorptive transcytosis can also act as a mechanisms of BBB transfer. Future investigations of modified IRAs taking into consideration acylation, size, and absolute charge, may enhance BBB penetration.

Within 1 h of iv injection, one of the IRAs tested here (tirzepatide) and three of those we tested earlier (liraglutide, semaglutide, Peptide 19).^50^ showed little evidence of entering the brain. This may reflect in part the sensitivity of our method using small amounts of radiolabeled peptides given the results of a recent study on semaglutide. That study used a novel liquid chromatography-tandem mass spectroscopy (LC-MS/MS) method to measure brain levels of semaglutide in adult male Sprague-Dawley rats injected iv 1 hour earlier with about 266 mg of the unlabeled drug per animal. Detectable amounts of semaglutide were found in the brain, especially the hypothalamus, but the absolute levels of the drug were very low with means of 12.0 ng/g in the brain as a whole, 12.6 ng/g in the cerebellum, 27 ng/g in the hypothalamus, and only 7.7 ng/g in the cerebrum (= cerebral cortex + striatum + amygdala + olfactory bulb).^75^ However, as this study did not perform vascular wash-out prior to brain collection and because there is about 1% plasma present in the brain, the brain values of semaglutide reported reflect, at least in part, contamination from blood.

Our results and the LC-MS/MS study just described are nevertheless consistent with the view that IRAs like liraglutide, semaglutide, and tirzepatide can slowly enter the brain by the extracellular pathways traversing cranial tissues where the BBB is weak or absent (e.g., circumventricular organs, nasal epithelium, and large subarachnoid blood vessels).^64^ Such extracellular pathways are used by intravenously administered antibodies, erythropoietin, and soluble receptors entering the brain in amounts able to exert biological effects.^64,76^ There is at least one indication that IRAs not detectably infiltrating the brain 1 h after iv injection (presumably due to an inability to cross the BBB) may still infiltrate the brain slowly via the extracellular pathways, namely that liraglutide, semaglutide, and tirzepatide bind serum albumin, which is known to enter the brain very slowly via such pathways.^64,77^ This feature of albumin was used to test brain infiltration of liraglutide, semaglutide, and tirzepatide over more than the hour used to assess transport across the BBB. We first confirmed that B/S ratios for iv administered ^99m^Tc-albumin were greater at 6 h than at 10 min or 1 h, consistent with albumin’s entry into the brain.^77^ When we compared delta B/S levels of ^125^I-labeled liraglutide, semaglutide, and tirzepatide after correction for serum degradation, greater levels of the labeled IRAs were found at 6 h compared to 10 min and 1 h indicating greater entry into the brain over the longer time period. Further analyses estimating the entry rate for each IRA over the 6 h period also suggested a reason for liraglutide’s inability to cross the BBB, specifically that it is reversibly bound to the luminal surface of the BBB and primarily present in the brain capillaries.^50^

As was the case for IRAs crossing the BBB in our earlier study,^50^ those crossing the BBB in the present study (albiglutide (BAF) and dulaglutide (BAF)) did not do so by a saturable process since the B/S ratios of their radiolabeled forms were unaffected by addition of 1 μg iv dose of the unlabeled IRA.

### 4.3. Relative brain vascular binding (V_r_) of IRAs

As can be seen in Figure 2C, the linear regression curves correlating delta B/S ratios to exposure time intersect the y-axis at values greater than 0. This value is equivalent to *V*_r_ since we have corrected for vascular space, and this value usually represents binding to receptors located on the luminal surface of brain endothelial cells. These results indicate a binding site for these peptide analogs on brain endothelial cells that could be GLP-1 R or GIPR. As exemplified by insulin and ghrelin, such binding sites can be functional receptors that influence brain endothelial cell functions that are unrelated to the transport of the peptide across the BBB.^69,78,79^

The *V*_r_ of IRAs in the present study was quite variable. Comparing the *V*_r_ of ^14^C-labeled DA5-CH to that of ^125^I-labeled albiglutide (BAF), dulaglutide (BAF), and tirzepatide, we found that DA5-CH, Model 1 (11.20 μl/g) vascular binding was far higher than that of all other IRAs studied. The *V*_r_ of albiglutide (BAF) (*V*_r_ = 2.90 μl/g), however, was itself much greater than tirzepatide (*V*_r_ = 0.03 μl/g) and dulaglutide (*V*_r_ = −0.15 μl/g). As discussed above, DA5-CH may have a rapid uptake phase followed by an early onset of equilibrium. Such a combination of uptake features can artificially elevate the *V*_r_. On average, the IRAs we tested previously.^50^ exhibited a *V*_r_ = 1.3 μl/g without a clear difference between single and dual IRAs. Comparing *V*_r_ values for all the IRAs we have studied under the same conditions, values between −0.15 and 2.90 μl/g do not predict if an iv-administered IRA gains access to the brain. Binding to receptors on brain endothelial cells could nevertheless have functional significance judging from functional effects of insulin on brain endothelial cells.^79–83^

It should be kept in mind, however, that we still lack direct evidence that IRAs interact with endothelial cells in the brain. This is apparent from the study of^73^ which reports that semaglutide does not interact with endothelial cells in the arcuate nucleus of the mouse hypothalamus, where endothelial cells lack GLP-1 R. It remains unknown if brain endothelial cells express GLP-1 R or GIPR protein. RNA-sequencing studies suggest very little to no RNA presence in brain endothelial cells of these two receptors.^84^ The ability of dual IRAs to interact with related receptors on endothelial cells is further limited by the finding that, where studied, they show only negligible binding of GLP-2 or glucagon receptors.^72,85^

### 4.4. Relative total influx of IRAs into brain and its parenchyma

To better identify the amount of IRA reaching the brain, we calculated the percentage of the injected dose that was taken up per gram of brain tissue (%Inj/g). Our studies use sub-micromolar levels of IRA to reduce the likelihood of physiological changes or therapeutic effects, allowing us to quantitatively measure the blood-to-brain transport rates with radioactivity. Albiglutide (BAF) peaked in brain at 15 min (0.36%Inj/g) and then declined until 60 min. Dulaglutide (BAF) exhibited a more sigmoidal uptake with levels elevated at 10 min (0.20%Inj/g) and then gradually increasing to 60 min (0.33%Inj/g).

We considered the possibility that the blood-to-brain transport rate for each IRA may be affected by its sequestration and degradation in brain endothelial cells. To evaluate this, we performed capillary depletion which separates capillary and parenchymal fractions of the brain. For those IRAs crossing the BBB, the majority of the radioactive label was present in brain parenchyma at 15 min. This indicates that these IRAs are not retained by brain endothelial cells but enter brain tissue (parenchyma) itself.

Exenatide is the IRA with the greatest %Inj/g by 60 min (0.56%Inj/g), which is continuing to rise at that time point. Albiglutide (BAF) and dulaglutide (BAF) as measured in this study are the next highest. These values are nearly threefold greater than most other hormones or drugs known to cross the BBB and affect insulin signaling.^86^

A peptide’s *K*_i_, %Inj/g, and %Inj/g AUC are important in determining the degree to which a peptide enters the brain. Calculating the AUC for each %Inj/g curve allows us to compare cumulative brain exposure for each IRA over time independent of differences in peak uptake time and rate of clearance, which is important for translational purposes. For example, albiglutide (BAF) peaks early (by 15 min) and slowly declines afterward while exenatide gradually increases with time, surpassing albiglutide (BAF) %Inj/g values at the 30 min time point. Due to the differences in these curves, the AUC provides us with an integrated value of brain exposure. %Inj/g values for DA5-CH (Model 2) capture this rapid equilibrium exposure in which levels peak at 1 min and decline to the minimum value by 5 min. Albiglutide (BAF) and exenatide have equivalent AUCs, suggesting that the same amount of these IRAs reaches the brain within 1 hour. These AUCs are roughly sevenfold greater than the IRA with the lowest AUC (Peptide 21).

The rank order of IRAs showing significant brain uptake 1 h after iv injection with respect to AUCs is similar, but not identical to the rank order of their K_i_’s (see Figures 5b and 3b). Less similar is the rank order of IRAs for uptake into brain parenchyma defined as the percent reaching the brain that was in its parenchyma vs. its capillaries. The rank order of parenchymal uptake was specifically DA4-JC (89.7%) = albiglutide (BAF) (89.4%) > DA3-CH (86%) > exenatide (84.2%) > Peptide 17 (73.2%) > lixisenatide (66.8%) > dulaglutide (BAF) (61.8%) > DA5-CH (57.6%) > Peptide 21 (43.6%) > liraglutide (28.7%). It should be noted, however, that the same four IRAs were among the five IRAs ranked highest for both Ki and AUC (i.e., albiglutide (BAF), dulaglutide (BAF), exenatide, and DA4-JC). With the exception of dulaglutide, the same peptides were also the highest ranked for parenchymal uptake.

### 4.5. Summary and conclusions

In this and our earlier study,^50^ we have collectively investigated brain uptake pharmacokinetics of 13 different peptide IRAs, including all those that are FDA-approved (albiglutide (BAF), dulaglutide (BAF), exenatide, liraglutide, lixisenatide, semaglutide, and tirzepatide), BAF peptides of albiglutide and dulaglutide, all the experimental dual IRAs that are equivalently potent activators of GLP-1 R and GIPR (Peptides 17, 18, 19, and 21 of Finan and Ma et al. [2013]), and all dual IRAs found therapeutic in animal models of AD and/or PD by Christian Hölscher.^38^ (DA1-JC, DA3-CH, DA4-JC, and DA5-CH). The two sets of dual IRAs just noted are not mutually exclusive. DA1-JC and DA3-CH are, respectively, Peptides 19 and 18 of Finan-Ma et al. (2013), and DA4-JC and DA5-CH are variants of Peptide 17 of Finan and Ma et al. (2013) with different C-terminal extensions designed to enhance BBB transport.^38^

We investigated the BBB transport rate of IRAs within 1 h (*K*_i_), total accumulation of brain uptake over 1 h (AUC), and the ability of longer-lasting IRAs to enter the brain over a 6 h period. The rate of transport specifies how quickly the IRA enters the brain, while %Inj/g levels specify the amount of IRA reaching the brain with the AUC specifying the %Inj/g integrated over time.

There was more than a 27-fold difference in brain entry rates (*K*_i_) among the 9 IRAs that readily crossed the BBB, which shows the great variability in BBB transport rates of these drugs. The rank order among these 9 IRAs with significant BBB transport within 1 h of iv injections was: DA5-CH, Model 2 >> albiglutide (BAF) > dulaglutide (BAF) >> DA4-JC > exenatide > DA3-CH (Peptide 18) > Peptide 17 > lixisenatide > DA5-CH, Model 1 ≫ DA2 (Peptide 21). Our protocol did not detect brain influx within 1 h of iv injections of radiolabeled DA1-JC, liraglutide, semaglutide, or tirzepatide, which indicates that these IRAs do not detectably cross the BBB within this time frame. Modeling indicates two mechanisms relating to BBB permeability: i) acylation greatly retards BBB penetration and ii) transmembrane diffusion as determined by lipid solubility and molecular weight influences the rate at which non-acylated IRA’s cross the BBB.

Despite the differences in rank order of IRAs for *K*_i_, %Inj/g AUC, and parenchyma uptake, the same four peptides were among the IRAs ranked highest for both *K*_i_ and %Inj/g AUC (i.e., albiglutide (BAF), dulaglutide (BAF), exenatide, and DA4-JC). With the exception of dulaglutide, these peptides were also the highest ranked for parenchymal uptake. DA5-CH was considered of special interest because of its potentially very fast brain uptake.

Due to the ability to cross the BBB, albiglutide (BAF), dulaglutide (BAF), exenatide, DA4-JC and possibly DA5-CH are higher priority IRAs for testing as AD and PD therapeutics. While all five of these IRAs are known or likely to be safe for human use given FDA approval of all but one (DA5-CH) of the five IRAs just listed,^61,87^ additional preclinical research is needed to determine which are most promising for clinical trials. This requires more extensive testing of albiglutide (BAF) and dulaglutide (BAF) on animal models of AD and PD, as well as testing all five IRAs on ex vivo preparations of brain tissue from AD and PD cases to determine, where possible, effects on disease modifying processes (e.g., brain insulin resistance).

Our continued investigation of brain penetration of peptide IRAs with structural variation aids in the design of new IRAs to achieve greater rates of brain uptake, allowing for greater therapeutic effects.

## Data Availability

All data were analyzed in Excel and placed in Graph Pad Prism 9.0 for graphing and data analysis. The raw data are made available upon reasonable request.

## Category best describing this work

Pharmacology and pharmacy: incretin receptor agonists.
